# Multimodal indicators of non-functional overreaching in NCAA Division I American football athletes: evidence for role-specific physiological adaptation

**DOI:** 10.3389/fphys.2026.1818629

**Published:** 2026-07-21

**Authors:** Mackenzie M. Aychman, Kelsey Vinson, Noor Tasnim, Jorge Perez, Georgia E. Hodes, Julia C. Basso

**Affiliations:** 1 Department of Human Nutrition, Foods, and Exercise, Virginia Tech, Blacksburg, VA, United States; 2 Department of Sports Science, Football, Virginia Tech, Blacksburg, VA, United States; 3 Graduate Program in Translational Biology, Medicine, and Health, Virginia Tech, Roanoke, VA, United States; 4 Post-baccalaureate Research Education Program, Virginia Tech, Blacksburg, VA, United States; 5 School of Neuroscience, Virginia Tech, Blacksburg, VA, United States; 6 Center for Health Behaviors Research, Virginia Tech Carilion, Roanoke, VA, United States

**Keywords:** countermovement jump, immune system, reactive strength index-modified, sleep, stress

## Abstract

**Introduction:**

The purpose of this study was to characterize longitudinal patterns associated with non-functional overreaching (NFOR) in NCAA Division I American football athletes across a competitive season using salivary biomarkers, neuromuscular performance measures, and recovery metrics, with exploratory analyses by team role.

**Methods:**

Thirty-nine male athletes (age 20.4 ± 1.75 years) were monitored across a 15-week competitive season. Saliva samples collected pre-, mid-, and post-season assessed cortisol, testosterone, interleukin-6 (IL-6), and tumor necrosis factor–alpha (TNF-α). Neuromuscular performance was evaluated using countermovement jump metrics (jump height, normalized peak power and force, reactive strength index–modified [RSImod]) and PlayerLoad, while recovery was tracked via twice-weekly self-reports of sleep quality and muscle soreness. Associations among biomarkers, recovery, and performance were examined by starting status and position group; statistical significance was set at p ≤ 0.05.

**Results:**

Distinct role-dependent patterns emerged. Among starters, higher testosterone-to-cortisol ratios were associated with lower soreness and better sleep quality, while higher IL-6/cortisol ratios were associated with higher RSImod values. Non-starters showed patterns consistent with greater physiological strain, with higher IL-6/cortisol ratios associated with poorer recovery despite lower external loads. Mid-season declines in countermovement jump performance coincided with biomarker changes suggestive of accumulated fatigue.

**Discussion:**

Overall, both starters and non-starters exhibited patterns consistent with NFOR, though physiological signatures differed by role. Salivary biomarker ratios, particularly IL-6/cortisol, alongside neuromuscular measures such as RSImod may provide useful, non-invasive indicators of fatigue-related stress and support role-specific recovery strategies in applied sport settings.

## Introduction

**Figure 1:**
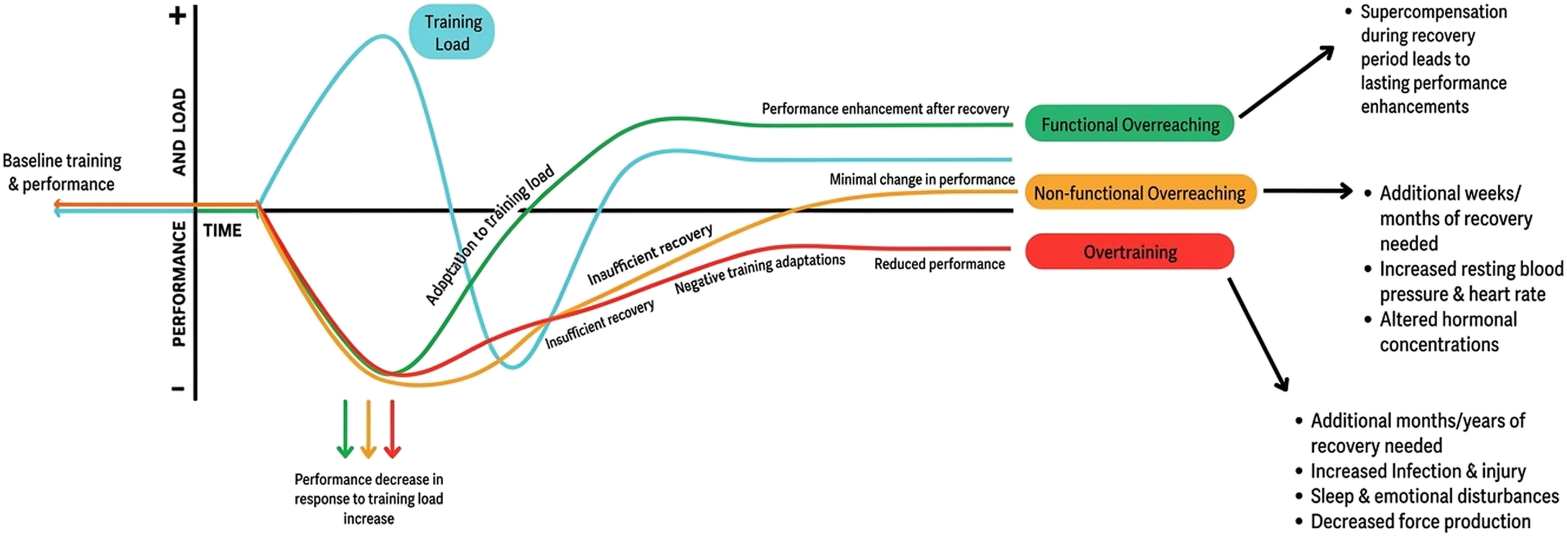
Overreaching and overtraining continuum. Varying responses to increased training load and insufficient recovery across time and training demands.

American football is one of the most physically demanding and popular sports in the United States, with athletes frequently exposed to high-impact collisions, demanding weekly schedules, and intense performance pressure (Edwards et al., 2018). Collegiate athletes may be especially vulnerable to exceeding their recovery capacity due to pre-season training demands, within-team competition for starting roles, and aspirations to reach the professional level (Hamlin et al., 2019; Lopes Dos Santos et al., 2020). This environment increases susceptibility to conditions such as non-functional overreaching (NFOR) and, in more severe cases, overtraining syndrome (OTS), both of which are marked by fatigue, performance decline, and incomplete recovery. These maladaptive states sit at one end of an overreaching-overtraining continuum that begins with functional overreaching (FOR), in which short-term performance decrements are intentionally induced and followed by supercompensation and lasting performance gains; NFOR and OTS occur when accumulated load exceeds recovery capacity (**Figure 1**) (Kreher and Schwartz, 2012; Myrick, 2015).

In team-sport athletes, NFOR is typically driven by repeated high workloads with inadequate recovery, resulting in hormonal, neuromuscular, and inflammatory dysregulation (Meeusen et al., 2013). While no single marker can definitively diagnose NFOR or OTS, salivary concentrations of interleukin-6 (IL-6), tumor necrosis factor-alpha (TNF-α), testosterone, and cortisol have been associated with fatigue-recovery states (Myrick, 2015; Brel et al., 2023). Cortisol reflects catabolic stress, while testosterone supports anabolic processes; their ratio (i.e., the TC ratio) serves as a marker of training balance (Banfi and Dolci, 2006). Interleukin-6 and TNF-α are pro-inflammatory cytokines that increase in response to physical stress and immune activation (Gokhale et al., 2007). A disproportionately elevated IL-6 to cortisol ratio may signal disrupted immunoendocrine feedback and systemic fatigue (Hackney and Walz, 2013). When interpreted alongside neuromuscular measures such as countermovement jump performance and PlayerLoad, these biomarkers provide non-invasive insights into physiological readiness and maladaptation.

Importantly, exposure to training and competition stressors is not uniform within team-based sports. In collegiate American football, starting status and positional role strongly influence both external workload and recovery opportunity across the season (Jagim et al., 2021). Starters are typically exposed to sustained, high-volume competition loads, whereas non-starters may experience more irregular training demands, characterized by fluctuating workloads, limited competitive exposure, and inconsistent recovery rhythms (Wellman et al., 2017). Such disparities may differentially influence physiological regulation, even when overall training volume appears lower (Gabbett, 2016). As a result, role-specific patterns of fatigue and recovery may emerge that are not captured by workload metrics alone, underscoring the importance of stratified analyses when interpreting athlete monitoring data.

Although physiological monitoring has been examined in American football athletes (McGuigan, 2017; Kraemer et al., 2013), relatively fewer studies have integrated endocrine, neuromuscular, and perceptual indicators longitudinally across a competitive season. Given the multifactorial nature of non-functional overreaching (NFOR) and the absence of a single diagnostic marker, examining convergent patterns across endocrine, inflammatory, neuromuscular, and perceptual domains may provide more ecologically valid insight into fatigue-related maladaptation in applied sport settings (Alba-Jiménez et al., 2022). Therefore, this study aimed to assess indicators of NFOR by integrating salivary biomarkers, neuromuscular performance, and subjective recovery, with particular focus on differences by starting status and position group. Findings from this study may inform individualized recovery strategies and performance management approaches that reduce the risk of maladaptation across the competitive season. From a strength and conditioning perspective, identifying whether fatigue indicators differ by player role may help practitioners tailor recovery strategies and optimize training load distribution. We hypothesized that starters and non-starters would demonstrate distinct biomarker, performance, and recovery profiles reflecting role-specific physiological adaptation across the season.

## Methods

### Subjects

Participants (n = 39, age range 18–25) were NCAA Division I American football athletes recruited prior to the 2024 pre-season. Athletes were recruited via convenience sampling from the active 2024 roster of a single NCAA Division I football program (Virginia Tech); active roster status was the sole eligibility criterion, and all consenting players were enrolled. No additional inclusion or exclusion criteria were applied. Ethical approval was obtained from the Virginia Tech Institutional Review Board as well as the Virginia Tech Athletics Department Institutional Review Board, and all participants provided written informed consent.

### Experimental approach to the problem

This study employed a longitudinal observational design to examine indicators of NFOR across a competitive NCAA Division I American football season. Given the multifactorial nature of NFOR, an integrated monitoring approach was used combining endocrine and inflammatory biomarkers, neuromuscular performance metrics, and subjective recovery measures. Salivary biomarkers were selected to provide non-invasive insight into immunoendocrine responses to training stress, while countermovement jump performance and PlayerLoad were included as established indicators of neuromuscular fatigue and external workload. Self-reported sleep quality and soreness were assessed to capture perceptual aspects of recovery. Because training exposure varies substantially by team role, analyses were stratified by starting status and positional group to evaluate whether physiological and performance responses differed according to competitive demands. This approach enabled evaluation of convergent indicators of fatigue and adaptation in a real-world athletic setting consistent with applied strength and conditioning practice.

### Procedures

Recovery metrics included self-reported soreness and sleep quality, assessed twice weekly prior to morning training using 11-point Likert scales administered through a secure online platform. These scales were developed by the Virginia Tech Athletics Department based on the Recovery-Stress Questionnaire for Athletes (Kellmann and Kallus 2001) and the wellness survey from Ihsan et al. (2017). The sleep quality slider was anchored 0 = “Well Rested,” 5 = “Somewhat Well Rested,” 10 = “Completely Exhausted,” and the soreness slider 0 = “No Soreness,” 5 = “Somewhat Sore,” 10 = “Completely Sore”; surveys were delivered twice weekly via the Smartabase athlete-monitoring platform on standardized mornings prior to training. External workload was quantified using Catapult Athlete Monitoring System GPS/LPS units (Catapult Sports, Melbourne, Australia) worn over the upper thoracic spine between the scapulae in a non-restricting vest or shoulder-pad pocket. Units were unique to each athlete to minimize between-unit variability and were activated immediately before warm-up and deactivated at the cessation of each training session and competition; athletes did not wear units outside of organized training and games. Data were downloaded after each session and PlayerLoad was calculated in Catapult OpenField software as the summed multi-directional acceleration vector divided by a scaling factor of 100. Neuromuscular performance was assessed weekly via countermovement jump testing using VALD ForceDecks dual force plates, with reactive strength index–modified (RSImod) calculated as jump height divided by time to takeoff based on the average of three trials. Contextual variables of position group and starting status were recorded.

Saliva samples (1 mL) were collected via passive drool in a private space in the morning, immediately prior to morning training at three standardized timepoints: pre-season (week 0), mid-season (week 7), and post-season (week 15). Sampling was scheduled within the same pre-training window at each timepoint to control for diurnal hormonal variation. Participants were instructed to refrain from eating or drinking for at least 30 minutes prior to each collection. Samples were immediately frozen at –80 °C and later assayed in duplicate for biomarker concentrations (IL-6, TNF-α, cortisol, and testosterone) using enzyme-linked immunosorbent assays (ELISA; Salimetrics, ThermoFisher, and Abcam).

### Statistical analyses

Sample size was determined by an *a priori* power analysis using G*Power 3.1, F tests, ANOVA: repeated measures, within factors, effect size f=0.25, power=0.8, 1 group, 3 measurements (based on biomarker testing), alpha=0.0125 (Bonferroni correction for 4 biomarkers), correlation among repeated measures of 0.5, and non-sphericity correction ϵ of 1, resulting in a sample size of n=38 (Faul et al., 2009).

Although sample size was informed by *a priori* power estimation for repeated biomarker measures, all analyses were conducted in an exploratory, applied framework consistent with longitudinal athlete monitoring research. Outlier values were winsorized prior to analysis to reduce the influence of extreme values while retaining all observations in this applied dataset, with extreme values adjusted to the 5th and 95th percentile thresholds. Linear mixed-effects models were used to examine relationships between biomarkers, recovery, and performance metrics, along with relevant covariates related to season timepoint and team role. Athlete ID was included as a random intercept to account for repeated measures. Separate mixed-effects models were fitted for each outcome (soreness, sleep, PlayerLoad, RSImod, jump height, peak power, and peak force), with predictors including biomarker concentrations, recovery scores, team role (starting status and position type), and interactions. Models were fitted using the lme4 package in R, with p-values derived using the lmerTest package (Kuznetsova et al., 2017). Bootstrapping was used to generate 95% confidence intervals. All models were interpreted descriptively, with emphasis on observed associations rather than causal inference. All analyses were conducted in R (version 4.3.1) (R Core Team and Others, 2013). All statistical analyses and subsequent interpretation are consistent with CHAMP (Mansournia et al., 2021). A p value of ≤0.05 was utilized to determine statistical significance.

## Results

Thirty-nine NCAA Division I American football athletes (mean age = 20.4 ± 1.75 years; height = 189 ± 6.58 cm; body mass = 112 ± 21 kg (246 ± 46 lb)) participated in the study, with 59% classified as starters. Based on positional and anthropometric characteristics, participants were categorized into three groups: Big (offensive and defensive linemen; n = 21), Big Skill (linebackers, tight ends, and cornerbacks; n = 5), and Skill (wide receivers, quarterbacks, and specialists; n = 13). Results are organized by recovery and performance outcomes (**Table 1**), with emphasis on group-specific and longitudinal patterns observed across the competitive season.

**Table 1:** Role of biomarkers by interaction.

Biomarker	Interaction context	Effect direction	Statistical significance
Testosterone	Soreness (Big Skill)	↓ Soreness	p = 0.033
Testosterone	Sleep (Starter)	↑ Sleep Quality	p = 0.006
TC Ratio	Soreness (Starter)	↓ Soreness	p < 0.001
TC Ratio	Sleep (Starter)	↓ Sleep Quality	p = 0.002
IL-6/Cortisol Ratio	Soreness (Big)	↓ Soreness	p = 0.036
IL-6/Cortisol Ratio	Soreness (Skill)	↓ Soreness	p = 0.033
IL-6/Cortisol Ratio	Sleep (Starter)	↑ Sleep Quality	p < 0.001
IL-6	RSImod (Starter)	↑ RSImod	p < 0.001
IL-6	RSImod (Non-starter)	↓ RSImod	p < 0.001
IL-6	Normalized Peak Power × Soreness	↓ Power	p < 0.001
TNF-alpha	Normalized Peak Power	↑ Power	p < 0.001
TNF-alpha	Normalized Peak Power × PlayerLoad	↓ Power at High Load	p < 0.001

### Recovery outcomes

#### Soreness

**Figure 2:**
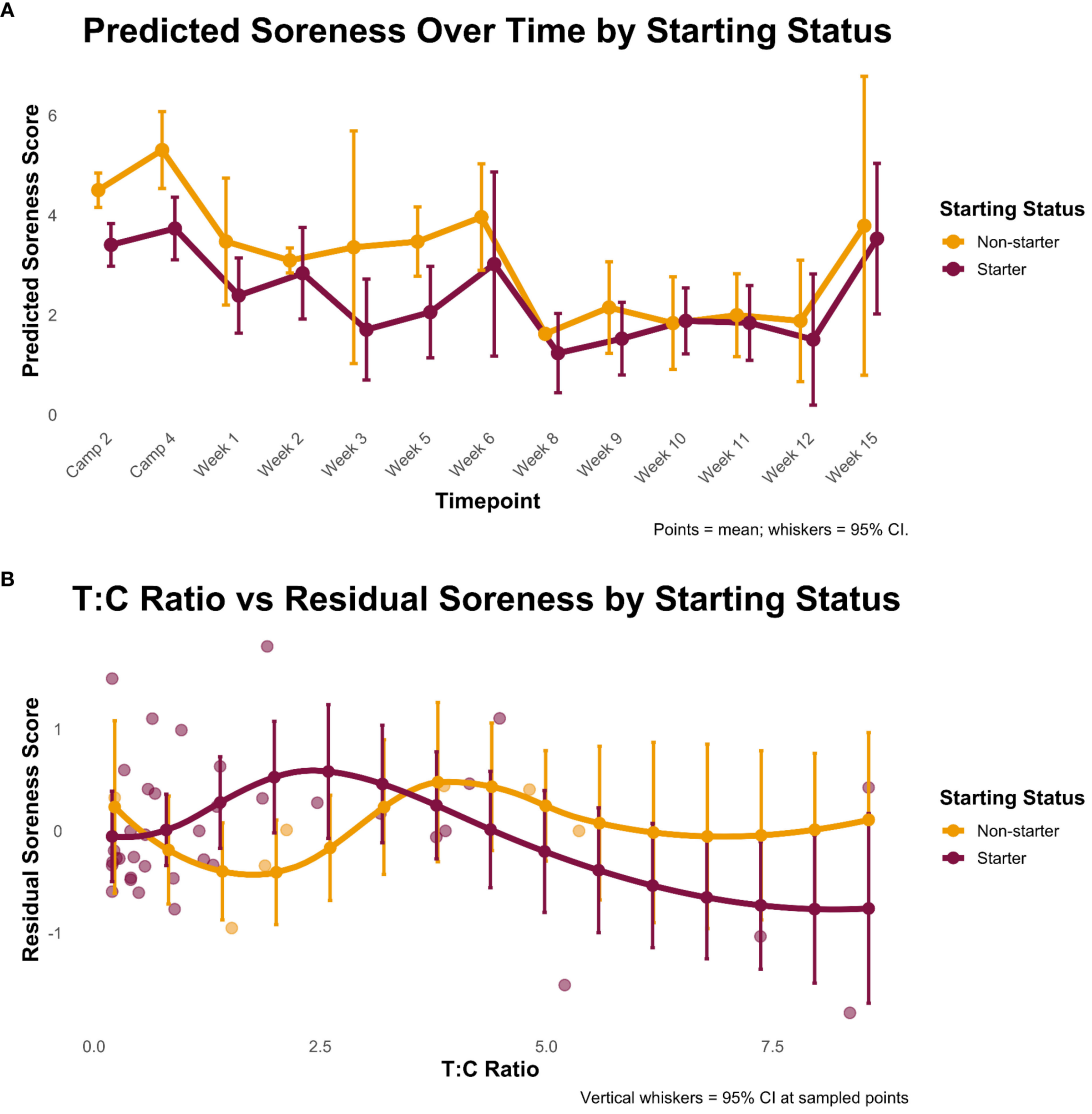
**(a)** Weekly soreness by starting status. Fitted model predicted soreness scores over time for starters and non-starters, measured across pre-season camp and weekly in-season timepoints. Non-starters consistently reported higher soreness than starters, with error bars representing variability (± 1 SE). “Camp 2” and “Camp 4” denote the second and fourth weeks of the pre-season training camp preceding Week 1 of the regular competitive season; the pre-season (Week 0) saliva sample reported in the Methods was collected during this pre-season camp period. **(b)** Residual soreness by TC ratio by starting status. Relationship between testosterone-to-cortisol (T:C) ratio and residual soreness scores for starters and non-starters. Solid lines represent LOESS-smoothed fits, with vertical whiskers denoting 95% confidence intervals at sampled T:C ratios. Non-starters show greater residual soreness than starters at higher T:C ratios, suggesting poorer adaptation to training stimuli.

Soreness differed by starting status and sleep quality. Specifically, soreness was lower in starters compared to non-starters (β = –1.08, t(15.70) = –2.62, p = 0.019) and was higher in athletes reporting poorer sleep quality (β = 0.54, t(190.82) = 10.05, p < 0.001) (**Figure 2a**). In biomarker interaction models, higher testosterone-to-cortisol (TC) ratios were associated with lower soreness in starters (β = –1.33, t(43.59) = –5.54, p < 0.001) (**Figure 2b**). Associations between the IL-6/cortisol ratio and soreness varied by position group, with higher ratios associated with lower soreness in Big athletes (β = –0.01, t(20.36) = –2.24, p = 0.036) and Skill athletes (β = –0.07, t(22.95) = –2.28, p = 0.033). The IL-6/cortisol ratio also interacted with sleep quality, such that higher ratios were associated with lower soreness among athletes reporting poorer sleep (β = –0.004, t(16.04) = –5.20, p < 0.001). Across the season, soreness was significantly elevated during the pre-season compared to all subsequent weeks, with no significant differences observed across the competitive season.

#### Sleep quality

**Figure 3:**
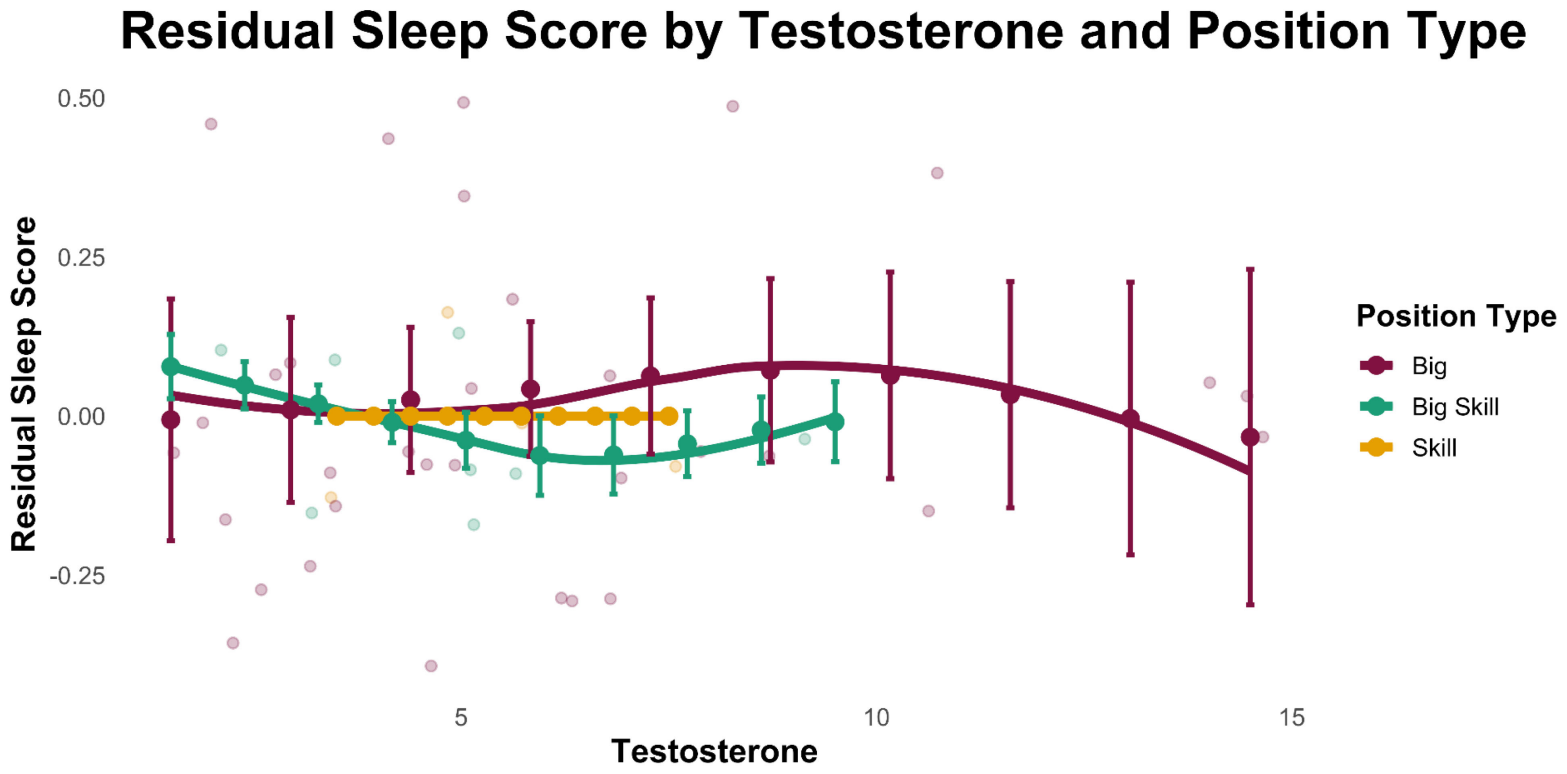
Residual sleep score by testosterone by position. Relationship between testosterone concentration and residual sleep scores across position types. Points represent individual observations, solid lines show LOESS-smoothed fits, and vertical whiskers denote 95% confidence intervals at sampled testosterone concentrations. Compared with skill and big-skill positions, players in big positions demonstrate greater variability in residual sleep across the testosterone range, as well as higher overall testosterone concentrations. Greater testosterone concentrations was associated with lower sleep quality in big skill athletes.

Poorer sleep quality was associated with greater soreness (β = 0.43, t(265.18) = 8.89, p < 0.001) and higher RSImod (β = 0.02, t(261.00) = 2.14, p = 0.033). In biomarker models, testosterone showed group-dependent associations with sleep quality, predicting poorer sleep in Big Skill athletes (β = 0.81, t(20.30) = 2.31, p = 0.032) (**Figure 3**) but better sleep in starters (β = –0.56, t(45.14) = –2.91, p = 0.006). The testosterone-to-cortisol (TC) ratio also exhibited group-specific relationships with sleep quality, predicting better sleep in Big (β = –1.45, t(37.81) = –2.74, p = 0.009) and Big Skill athletes (β = –2.14, t(14.69) = –2.37, p = 0.032), but poorer sleep in starters (β = 1.03, t(44.73) = 3.24, p = 0.002). Similarly, the IL-6/cortisol ratio predicted poorer sleep in Big, Big Skill, and Skill athletes (p < 0.001), but better sleep in starters (β = –0.96, t(24.52) = –4.21, p < 0.001). Across the season, sleep quality improved following the Week 8 bye compared to the Week 13 lead-up to the final game.

### Performance outcomes

#### PlayerLoad

**Figure 4:**
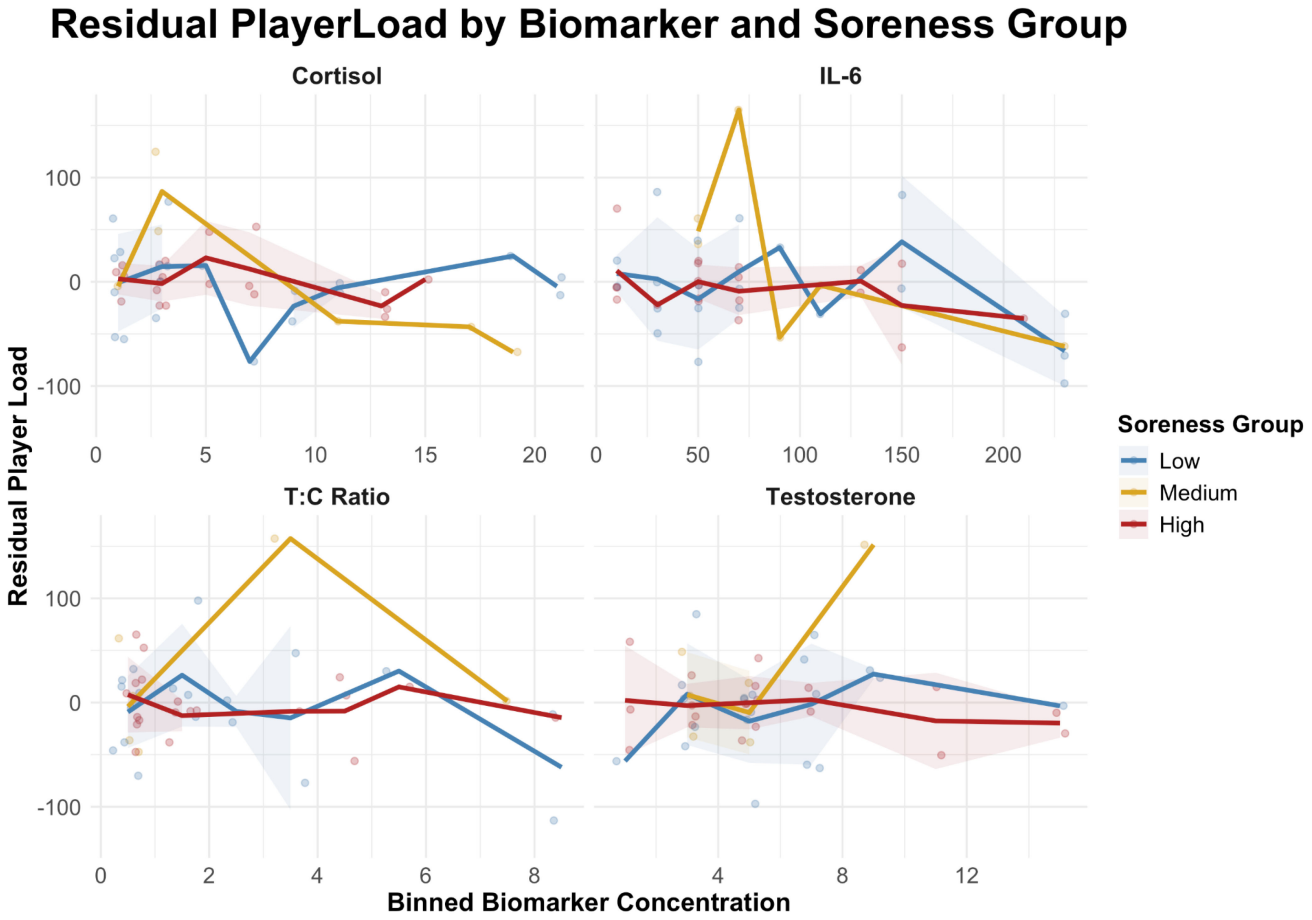
Residual PlayerLoad by biomarker and soreness. Residual PlayerLoad by biomarker concentration and soreness group. Line plots show the relationship between binned biomarker concentrations (Cortisol, IL-6, T:C Ratio, and Testosterone) and residual PlayerLoad after controlling for confounders. Data are grouped by self-reported soreness levels (Low = blue, Medium = yellow, High = red). Shaded regions represent variability within each group. Distinct patterns emerge across biomarkers, with medium soreness often associated with elevated residual load responses, particularly for IL-6, T:C Ratio, and Testosterone, while low and high soreness groups tend to track closer to baseline.

PlayerLoad, a marker of external workload during training, was lower in Skill athletes compared to Big athletes (β = –172.72, t(18.90) = –2.71, p = 0.014). In biomarker interaction models, soreness interacted with testosterone, cortisol, IL-6, the testosterone-to-cortisol (TC) ratio, and the IL-6/cortisol ratio to predict PlayerLoad (p < 0.001 for all interactions) (**Figure 4**). Across the season, PlayerLoad peaked during the pre-season and declined progressively throughout the competitive season, with the lowest values observed during the bye week and post-season.

#### Jump height

**Figure 5:**
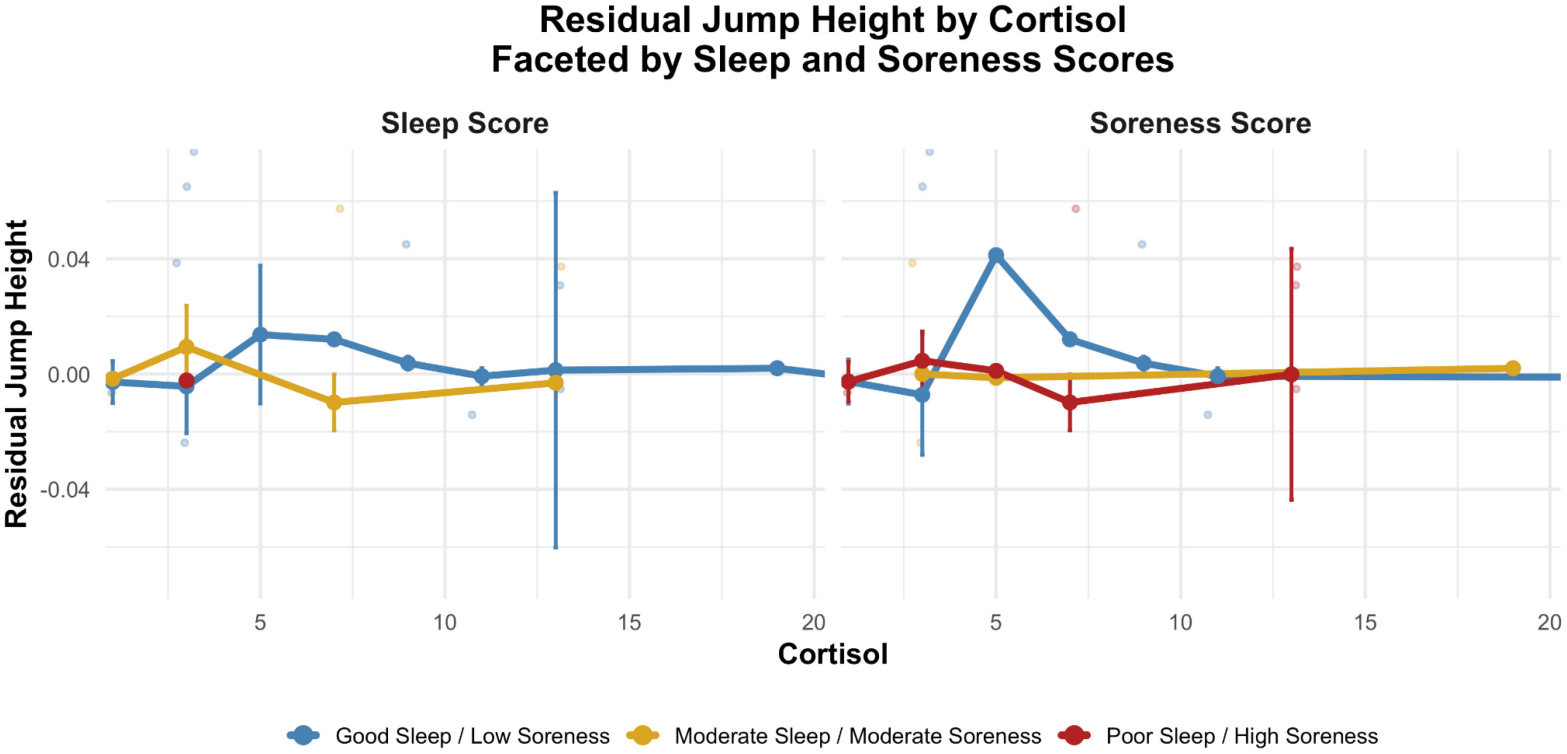
Jump height by cortisol by recovery. Points are individual observations and colored lines denote means across cortisol bins. Vertical whiskers show confidence intervals around each mean. Better recovery as demonstrated by lower soreness and better sleep quality is associated with higher jump heights across cortisol concentrations, while more soreness at the same concentration elicits lower heights.

Jump height was associated with interactions between recovery and hormonal markers. Poor sleep combined with higher cortisol was associated with higher jump height (β = 0.38, t(28.60) = 5.40, p < 0.001), whereas the interaction between soreness and cortisol was associated with reduced jump height (β = –0.21, t(29.98) = –4.73, p < 0.001) (**Figure 5**). Testosterone also interacted with soreness, with higher testosterone associated with greater jump height under conditions of increased soreness (β = 0.08, t(16.42) = 2.54, p = 0.02). The IL-6/cortisol ratio showed position-dependent associations with jump height, predicting lower jump height in Big and Big Skill athletes and higher jump height in Skill athletes. Across the season, jump height declined from the pre-season to mid-season and rebounded in the post-season.

#### Normalized peak power

Normalized peak power, measured during the countermovement jump test, was lower in athletes reporting greater soreness (β = –0.55, t(266.38) = –2.33, p = 0.021). TNF-α was positively associated with normalized peak power (β = 0.86, t(14.22) = 6.38, p < 0.001); however, its interaction with PlayerLoad was negatively associated with peak power (β = –0.683, t(27.83) = –5.97, p < 0.001). The interaction between IL-6 and soreness was also associated with reduced normalized peak power (β = –0.87, t(31.64) = –5.94, p < 0.001). Across the season, reductions in normalized peak power were most pronounced following Week 1.

#### Normalized peak force

Normalized peak force, measured during the countermovement jump test, was higher in Big Skill and Skill athletes compared to Big athletes (p < 0.05). Greater soreness was associated with slightly higher normalized peak force (β = 0.08, t(219.04) = 2.65, p = 0.009). Testosterone showed role-dependent associations with normalized peak force, predicting lower force in starters (β = –0.66, t(20.70) = –2.78, p = 0.011) but higher force in non-starters (β = 0.26, t(27.95) = 2.51, p = 0.018).

#### Reactive strength index-modified

**Figure 6:**
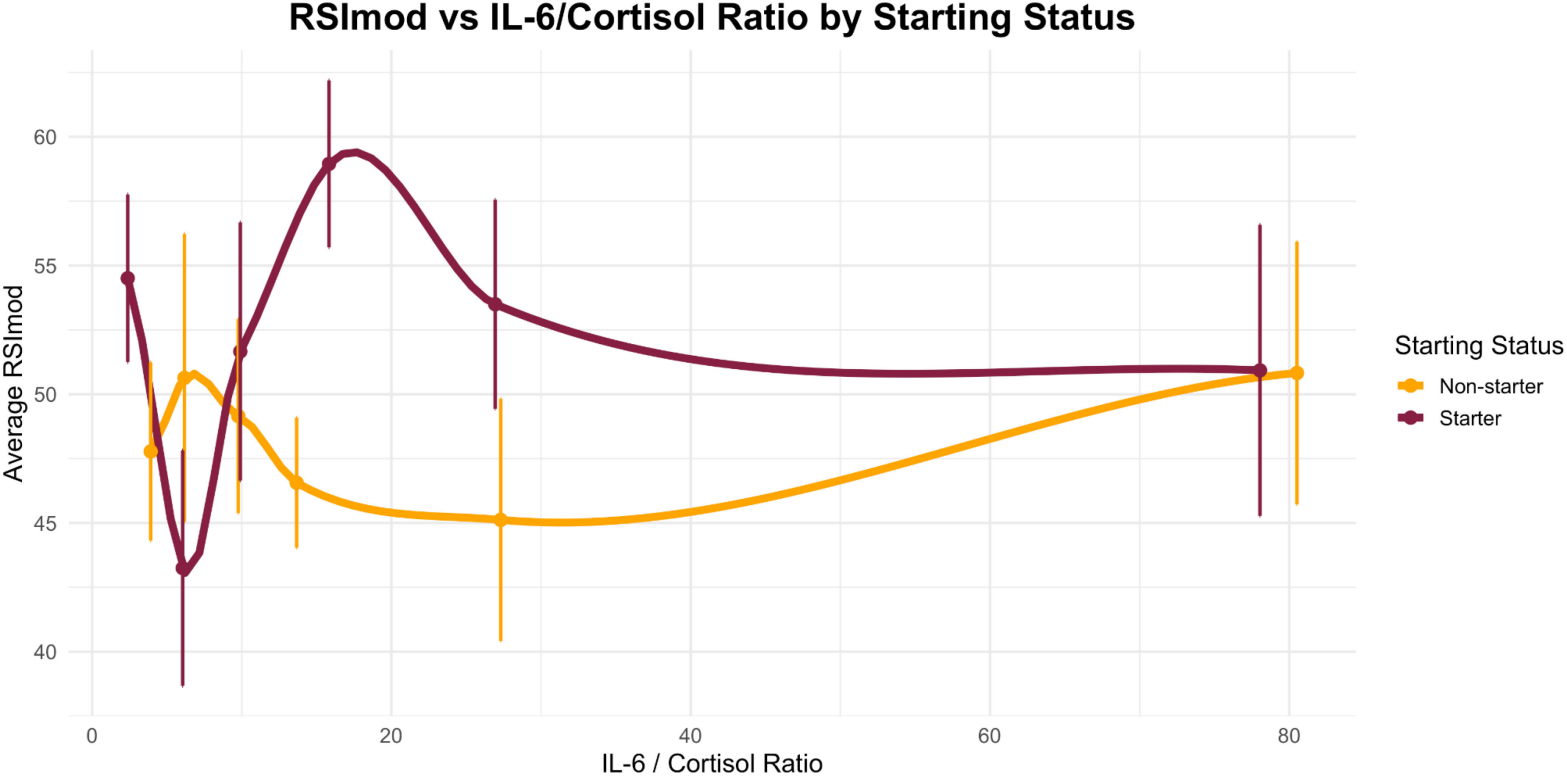
RSImod by IL-6/Cortisol ratio by starting status. Points are bin-level means of RSImod with vertical whiskers showing ±1 SE; smooth curves are LOESS fits for each group. Starters generally exhibited higher RSImod across the ratio range, with convergence at the highest ratio.

Reactive strength index–modified (RSImod), a marker of lower body explosiveness, was higher in Skill athletes compared to Big athletes (β = 16.76, t(22.59) = 3.69, p = 0.001). Interactions between IL-6 and recovery measures were associated with RSImod, with the IL-6 × sleep interaction associated with reduced RSImod (β = –0.93, t(23.22) = –5.80, p < 0.001) and the IL-6 × soreness interaction associated with greater RSImod (β = 0.76, t(24.00) = 5.07, p < 0.001). Testosterone was positively associated with RSImod (β = 0.19, t(19.63) = 3.40, p = 0.003), whereas a higher testosterone-to-cortisol (TC) ratio was associated with lower RSImod (β = –0.40, t(24.05) = –6.26, p < 0.001). The IL-6/cortisol ratio showed group-dependent associations with RSImod, predicting lower RSImod in non-starters but higher RSImod in starters and Skill athletes (p < 0.001) (**Figure 6**). Across the season, RSImod declined from the pre-season to mid- and late-season phases.

## Discussion

This study provides evidence that physiological stress, recovery, and performance adaptation differ meaningfully by player role across a competitive NCAA Division I American football season. Importantly, differences between starters and non-starters emerged as a primary discriminator of non-functional overreaching (NFOR), despite shared team environments and similar exposure to organized training. While indicators of NFOR were observed across the roster, starters and non-starters exhibited distinct patterns of biomarker activity, neuromuscular fatigue, and perceptual recovery, underscoring the need for role-specific monitoring strategies.

Previous work in collegiate American football has documented substantial physiological strain across the competitive season, but relatively few studies have integrated neuromuscular, biochemical, and perceptual measures longitudinally despite widespread practitioner use of such tools (Kraemer et al., 2009; Stone et al., 2019). The present findings extend this literature by suggesting that player role may represent an additional contextual factor influencing how endocrine, inflammatory, and neuromuscular signals should be interpreted in American football environments, particularly when distinguishing adaptive training responses from early signs of NFOR.

Indicators of NFOR were evident in both soreness and sleep outcomes, particularly when stratified by team role. Elevated soreness and disrupted sleep are established markers of overreaching (Kreher, 2016; Lastella et al., 2018), and in the present study, starters, who experience greater cumulative training and competitive load, exhibited role-specific patterns across these recovery measures. Together, these findings suggest differential recovery regulation by role, with starters demonstrating perceptual and physiological responses consistent with heightened seasonal stress exposure.

Although soreness was higher overall in non-starters, associations between soreness and hormonal recovery markers were more tightly coupled in starters, suggesting greater alignment between endocrine regulation and perceptual recovery under sustained training demands. Specifically, a higher testosterone-to-cortisol (TC) ratio was associated with reduced soreness in starters, consistent with a more favorable anabolic–catabolic balance during periods of cumulative load. In contrast, a higher IL-6/cortisol ratio, often linked to immune–endocrine activation, was associated with lower reported soreness in Big and Skill athletes, particularly when sleep quality was poor. While this pattern may appear counterintuitive, it may reflect perceptual blunting or fatigue masking, phenomena previously described in overreached or maladapted athletes (Meeusen et al., 2013; Coutts et al., 2021). In such states, subjective soreness may be attenuated despite accumulating physiological stress, or performance may be transiently maintained through compensatory mechanisms. Elevated soreness during the pre-season aligns with known peaks in training load and reinforces the importance of early-season monitoring to identify emerging fatigue prior to competitive intensification. Lower sleep quality was associated with greater soreness, but also coincided with elevated RSImod, suggesting that some athletes maintained neuromuscular output despite disrupted recovery, consistent with the fatigue-masking pattern noted above.

Biomarker associations with sleep quality further differed by team role. Testosterone was associated with better sleep in starters but poorer sleep in Big Skill athletes, whereas the testosterone-to-cortisol (TC) ratio showed the opposite pattern, predicting poorer sleep in starters yet better sleep in Big and Big Skill athletes. Notably, the IL-6/cortisol ratio was associated with poorer sleep quality in non-starters and across positional groups, but with better sleep in starters, underscoring that similar physiological signals may reflect adaptive or maladaptive processes depending on training status and role context.

Together, these patterns suggest that consistent exposure to high training loads, as experienced by starters, may be associated with more stable recovery regulation or stress tolerance compared with the irregular or subthreshold loading typical of non-starters. These findings align with prior work indicating that rotational or reserve athletes may face unique recovery challenges due to inconsistent training stimuli and reduced recovery structure (Kellmann et al., 2018; Ribeiro et al., 2024). Collectively, the results reinforce that team role mediates not only physical load exposure, but also biological and perceptual responses to stress, highlighting the importance of role-specific monitoring strategies that integrate biomarkers with subjective recovery measures for early identification of NFOR.

Performance outcomes across the season revealed patterns consistent with NFOR, particularly during high-load phases and when examined by player role. Metrics such as PlayerLoad, countermovement jump characteristics, and the modified RSImod captured not only changes in performance output, but also underlying neuromuscular fatigue and dysregulation. Examining these measures in relation to biomarker and recovery profiles allowed for a more nuanced characterization of adaptive versus maladaptive performance responses across the season.

External load, as captured by PlayerLoad, peaked during the pre-season and declined across the competitive cycle regardless of team role, reflecting a typical periodized training response (Malone et al., 2015; Gabbett, 2016). However, interactions between soreness and multiple biomarkers, including testosterone, cortisol, IL-6, and the IL-6/Cortisol ratio, revealed patterns consistent with hormonal compensation that may mask accumulating fatigue.

Specifically, higher testosterone in the context of elevated soreness was associated with lower PlayerLoad, whereas higher IL-6 or cortisol concentrations were associated with maintained or elevated PlayerLoad under comparable fatigue conditions. Although these biomarkers are physiologically interrelated, their convergent associations suggest some athletes may sustain external output through the stress-related compensatory mechanisms outlined above (Hackney and Walz, 2013). This pattern is consistent with an early, subclinical stage of NFOR that precedes overt performance decline. These findings underscore the value of integrating external load metrics with biomarker and recovery data to improve early detection of maladaptive training responses.

Neuromuscular performance showed sensitivity to cumulative fatigue and stress regulation, with jump height declining significantly mid-season, consistent with previously reported performance decrements during high-load phases in team sport athletes (Cormack et al., 2008). This decline was modulated by interactions involving cortisol, underscoring the context-dependent role of glucocorticoids in neuromuscular performance. Specifically, cortisol in the context of poor sleep was associated with higher jump height, potentially reflecting transient arousal or heightened sympathetic activation (Meeusen et al., 2013), whereas cortisol paired with elevated soreness was associated with reduced jump height, consistent with impaired force production under greater physiological strain.

IL-6/Cortisol effects on jump height were again role-specific. This ratio predicted reduced jump height in Big and Big Skill athletes, but higher jump height in Skill athletes. These divergent responses may reflect differences in positional demands and neuromuscular resilience, with lighter, speed-oriented players potentially better able to tolerate inflammatory or endocrine stress (McGuigan, 2017). Alternatively, elevated jump performance in this context may represent a compensatory overshoot, consistent with the fatigue-masking pattern described above (Taylor et al., 2016). Together, these findings highlight the importance of interpreting neuromuscular performance within both biomarker context and positional role in high-performance environments.

Normalized peak power and force provided additional insight into systemic neuromuscular strain under conditions of high external load. Elevated soreness and higher TNF-alpha concentrations were associated with reduced peak power at higher PlayerLoads, consistent with prior evidence linking pro-inflammatory signaling to impaired force production when recovery is inadequate (Suzuki et al., 1999; Smith, 2000).

Notably, testosterone and TNF-alpha exhibited role-dependent associations with normalized peak force. Testosterone was associated with reduced force output in starters but higher force output in non-starters. These divergent patterns may reflect differences in training adaptation and recovery regulation by role, with non-starters potentially experiencing relative overload due to inconsistent training stimuli, variable playing time, and less predictable recovery structure (Bourdon et al., 2017; Windt and Gabbett, 2017). This interpretation aligns with previous findings in American football and other team sports suggesting that reserve athletes may experience substantial physiological strain despite lower aggregate external loads, owing to misalignment between training exposure and recovery opportunity (Halson, 2014).

RSImod declined across the season and emerged as a sensitive indicator of neuromuscular fatigue and readiness, consistent with prior findings in team sport athletes (Ellis et al., 2022; Rebelo et al., 2024). IL-6/Cortisol ratios were associated with lower RSImod in non-starters, but higher RSImod in starters and Skill athletes, indicating that training status and positional role may influence whether physiological responses to stress reflect adaptive or maladaptive processes.

These patterns align with previous work showing that non-starters may experience dysregulated readiness due to irregular training stimuli and limited recovery structure, despite lower total workloads (Windt and Gabbett, 2017; Coutts et al., 2021). Together, these findings reinforce RSImod’s utility as a real-time marker of neuromuscular fatigue and highlight that both starters and non-starters may exhibit signs of NFOR, albeit through distinct physiological pathways related to load consistency, role demands, and immune–endocrine balance.

### Practical applications

Findings from this study highlight the importance of individualized monitoring and recovery strategies in team sport environments. Monitoring protocols should account for player role, as starters and non-starters demonstrated distinct physiological stress profiles despite comparable overall team workloads. Uniform training or recovery prescriptions may therefore overlook meaningful differences in adaptive versus maladaptive responses across athletes.

The IL-6/cortisol ratio emerged as a potentially informative indicator of systemic fatigue or maladaptation. Associations with sleep quality, perceived soreness, and neuromuscular performance suggest this biomarker may provide a non-invasive index of recovery status and early signs of overreaching when interpreted alongside other monitoring tools.

Neuromuscular performance metrics such as the RSImod offer additional real-time insight into athlete readiness. When integrated with biomarker data and self-reported recovery indices, RSImod may help identify both compensated fatigue (maintained performance despite physiological strain) and decompensated fatigue, supporting more informed training adjustments.

Finally, the results suggest that non-starters may be relatively under-monitored despite experiencing physiological stress related to irregular training stimuli and recovery structure. More consistent role-specific monitoring approaches that combine neuromuscular, biochemical, and perceptual measures may help optimize performance while reducing the risk of maladaptation across the full roster.

Several limitations should be acknowledged. First, the modest sample size restricts generalizability, particularly for subgroup analyses. Although position-specific stratification yielded informative trends, some subgroups were underpowered, limiting confidence in finer-grained comparisons. Given the modest sample size and number of predictors examined, some regression models may be susceptible to overfitting; therefore, findings should be interpreted as exploratory and hypothesis-generating rather than confirmatory. Second, while salivary biomarkers were collected at standardized timepoints, the absence of more frequent sampling limited our ability to capture short-term physiological fluctuations and acute recovery dynamics. Third, potentially influential external factors, including nutrition, psychological stress, and sleep behaviors, were not experimentally controlled and may have affected both biomarker expression and recovery outcomes. Fourth, participants were recruited via convenience sampling from a single NCAA Division I football program, which constrains external validity; replication across multiple institutions, divisions, and conferences is needed to confirm whether the role-specific patterns reported here generalize beyond this team and season. Fifth, sleep quality and soreness were captured exclusively via self-report and were not corroborated with consumer-grade sleep trackers, actigraphy, or heart-rate variability (HRV) monitoring. Modern wearable devices offer relatively low-cost access to objective indices of sleep architecture, sleep duration, and autonomic recovery, and direct comparison of subjective and objective sleep measures (along with HRV trends) is an important next step that would strengthen the biomarker–recovery–performance framework advanced here. Finally, given the observational design, causal relationships cannot be inferred; associations between biomarkers, performance metrics, and recovery indicators should therefore be interpreted cautiously.

### Conclusion

This study demonstrates that both starters and non-starters in NCAA Division I American football exhibit distinct indicators of NFOR, shaped by differences in role-specific training load, recovery patterns, and physiological regulation. Although starters experienced greater cumulative demands and soreness, their hormonal balance, particularly the testosterone-to-cortisol ratio, appeared to support short-term maintenance of neuromuscular performance. In contrast, non-starters showed evidence of physiological dysregulation despite lower external workloads, potentially reflecting inconsistent training stimuli and less structured recovery.

Salivary biomarkers, particularly the IL-6/cortisol ratio, provided insight into role-specific patterns of adaptation and maladaptation, demonstrating associations with both perceptual measures (soreness, sleep quality) and neuromuscular performance indices. These findings underscore the importance of individualized athlete monitoring strategies that integrate endocrine, inflammatory, and performance-based measures to facilitate early detection of overreaching and support more targeted recovery interventions across the competitive season. Collectively, these results suggest that athlete role, not simply workload magnitude, should be considered a central factor in performance monitoring and recovery programming in team sport environments.

## Data Availability

The raw data supporting the conclusions of this article will be made available by the authors, without undue reservation.
